# Phylogeographic Study of *Apodemus ilex* (Rodentia: Muridae) in Southwest China

**DOI:** 10.1371/journal.pone.0031453

**Published:** 2012-02-07

**Authors:** Qi Liu, Peng Chen, Kai He, C. William Kilpatrick, Shao-Ying Liu, Fa-Hong Yu, Xue-Long Jiang

**Affiliations:** 1 State Key Laboratory of Genetic Resources and Evolution, Kunming Institute of Zoology, Chinese Academy of Sciences, Kunming, Yunnan, China; 2 Graduate University of Chinese Academy of Sciences, Beijing, China; 3 Department of Biology, University of Vermont, Burlington, Vermont, United States of America; 4 Sichuan Academy of Forestry, Chengdu, China; 5 Interdisciplinary Center for Biotechnology Research, University of Florida, Gainesville, Florida, United States of America; University of York, United Kingdom

## Abstract

**Background:**

The Mountains of southwest China have complex river systems and a profoundly complex topography and are among the most important biodiversity hotspots in the world. However, only a few studies have shed light on how the mountains and river valleys promote genetic diversity. *Apodemus ilex* is a fine model for investigating this subject.

**Methodology/Principal Findings:**

To assess the genetic diversity and biogeographic patterns of *Apodemus ilex*, the complete cytochrome *b* gene sequences (1,140 bp) were determined from 203 samples of *A. draco/ilex* that were collected from southwest China. The results obtained suggested that *A. ilex* and *A. draco* are sistergroups and diverged from each other approximately 2.25 million years ago. *A. ilex* could be divided into Eastern and Western phylogroups, each containing two sub-groups and being widespread in different geographical regions of the southern Hengduan Mountains and the western Yunnan - Guizhou Plateau. The population expansions of *A. ilex* were roughly from 0.089 Mya to 0.023 Mya.

**Conclusions:**

Our result suggested that *A. ilex* is a valid species rather than synonym of *A. draco*. As a middle-high elevation inhabitant, the phylogenetic pattern of *A. ilex* was strongly related to the complex geographical structures in southwest China, particularly the existence of deep river valley systems, such as the Mekong and Salween rivers. Also, it appears that the evolutionary history of *A. ilex*, such as lineage divergences and population expansions were strongly affected by climate fluctuation in the Late Pleistocene.

## Introduction

*Apodemus* species are among the most common small rodents inhabiting woodlands and forests of the Palaearctic and Oriental Region [1], [2], [3], [4]. The genus has been subdivided into four subgenera, *Apodemus*, *Sylvaemus*, *Alsomys* and *Karstomys* [5] and comprises 20–22 extant species [2], [4], [6].

There are 12 extant *Apodemus* species in the Oriental Region [2], but only four forms are reported from the Eastern Trans-Himalayas [4], including *A. peninsulae*, *A. latronum*, *A. chevrieri* and the *A. draco* complex. The *Apodemus draco* complex include *A. draco*, *A. ilex*, and *A. orestes* and are distributed in mountain areas in China, Myanmar, and India [2]. These taxa have been treated as three valid species, subspecies of a single species, or synonyms of *Apodemus draco* in different taxonomic revisions [2], [3], [5], [7], [8]. The fossil records and phylogenetic analysis suggested an initial radiation of *Apodemus* in East Asia into a Japanese endemic (*A. argenteus*), a Nepalese endemic (*A. gurkha*) and the ancestral lineage of the remaining eastern Asian species (subgenus *Apodemus*) after a two-step radiation process associated with the recent tectonic movements that occurred 5–7 Mya (million years ago) and 2–3 Mya, respectively [9], [10], [11]. The phylogeography of *Apodemus* in the Far East of Asia showed extensive isolations within *Apodemus* species and could be linked to the presence of many biogeographic barriers such as mountains, rivers, seas, and deserts [1], similar to many other species [9], [12], [13], [14].

The Hengduan Mountains (i.e. the mountains of Southwest China) have the most complex river systems and a profoundly complex and dynamic geological history. The uplifting of the Himalayas and the Qinghai-Tibet Plateau and the successive alternation of glaciation and interglaciation in the Pliocene-Pleistocene contributed to the formation of natural geographical barriers and habitat heterogeneity [15]. This made it not only an important center of relic survival but also decisive evolutionary localities exist. Hence it is an excellent model system for biogeographic studies [16]. Previous studies of *Apodemus* considered this region to be the Pleistocene refugium or the radiation center for the East Asian *Apodemus* species [1], [8], [17], [18]. However, there has not been sufficient evidence from morphometric and molecular studies to describe the phylogenetic relationship between *A. draco* and *A. ilex*, especially the biogeography in the south of the Hengduan Mountains and the Yunnan-Guizhou Plateau.

In this study, the complete cytochrome *b* gene sequences (cyt-*b*) were determined from 203 samples of *A. draco/ilex* that were collected from southwest China, including the Hengduan Mountains and the Yunnan-Guizhou Plateau. Using phylogenetic and phylogeographical approaches, we examined the different revisions regarding the taxonomic status of *A. ilex*, the effect of the complex geological structures in the Himalayan regions on the genetic diversity of *A. ilex*, as well as the hypotheses of the biogeographic patterns and colonization history of *A. ilex*. In addition, a Bayesian method with a “relaxed” clock model [19] was applied to co-estimate the phylogenetic relationships and divergence times of *Apodemus*.

## Methods

### Ethics Statement

All animal samples were obtained following the regulations for the implementation of China on the protection of terrestrial wild animals (State Council Decree [1992] No. 13) and approved by Wildlife Protection Office, Yunnan and Sichuan Provincial Forestry Departments, China as well as the Ethics Committee of Kunming Institute of Zoology, Chinese Academy of Sciences, China.

### Sampling and sequencing

**Figure 1:**
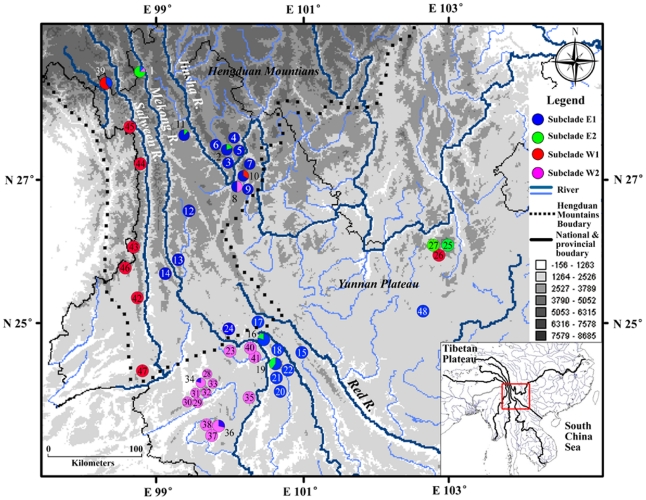
Samples of *A. ilex* used in this study. Numbers are corresponding to those in Table 1 and presented as pie-charts. Slice size proportional to the frequency of the subclades occurring in the site.

A total of 203 samples of *Apodemus draco/ilex* were collected from 51 localities in the southern Hengduan-Mountains and the Yunnan-Guizhou Plateau in China (Table S1, Figure 1). Specimens were identified based on the description of Thomas [20] and Barrett-Hamilton [21].

Total genomic DNAs were extracted from muscle or liver preserved in 95% ethanol at −20°C by using the phenol/proteinase K/sodium dodecyl sulphate method [22]. Mitochondrial cyt-*b* sequences (1,140 bp) were amplified with the universal primers of L14724 and H15915 [23]. The 50 µl PCR reaction contained 5 µl of 10X PCR buffer, 2 µl of 2 mM dNTP mixture, 2 µl of bovine serum albumin (1 mg/ml), 1 µl of 10 mM of each primer, 2.5 µl of 25 mM MgCl_2_, 1.25 U rTaq DNA Polymerase (Takara, Dalian, China) and approximately 100 ng total genomic DNA as template, and DNase/RNase free water diluted to a final volume of 50 µl. A touchdown PCR protocol [24] was used to prevent non-specific amplification, including an initial denaturation at 94°C for 10 min, 10 cycles of denaturation at 94°C for 60 s, annealing at 52.5°C but decreased by 0.5°C per cycle for 60 s, extension at 72°C for 60 s, followed by additional 20 cycles of denaturation at 92°C for 60 s, annealing at 47.5°C for 60 s, extension at 72°C for 60 s, and a final extension at 72°C for 10 min. PCR products were purified with UNIQ-10 spin column DNA gel extraction kit (Shengong, Shanghai, China) and sequenced from both directions with the same PCR primers in an automated DNA sequencer (ABI PRISM 3730) by using Big Dye terminator v3.1 in Tiangen Biotech CO., LTD (Beijing, China).

In addition, 34 cyt-*b* sequences from GenBank, including 4 sequences of *A. ilex*, 7 sequences of *A. draco*, and 23 sequences of *A. agrarius*, *A. alpicola*, *A. chevrieri*, *A. flavicollis*, *A. latronum*, *A. mystacinus*, *A. peninsulae*, *A. semotus*, *A. speciosus*, *A. sylvaticus A. uralensis*, *Mus musculus*, and *Rattus rattus* were included in analyses (Table S2).

### Phylogenetic and molecular divergence analysis

The DNA sequences were edited with Seqman and EditSeq (DNASTAR, Lasergene v7.1) and aligned with ClustalX v1.83 [25]. Genetic distance was calculated with MEGA v4.0 [26] with the Kimura 2-parameter (K2P) model [27].

We apply Bayesian inference (BI) and maximum likelihood (ML) to reconstruct phylogenetic relationships. We used RAxML v7.2.8 [28], [29] for ML analyses on the CIPRES Science Gateway v3.1 ([30] http://www.phylo.org). The data set was partitioned by codon. We used the GTRGAMMA model for each partition as recommended by RAxML and selected the novel rapid Bootstrapping algorithm [29] and ran 450 bootstrap replicates.

We used BEAST v1.5.4 for simultaneous Bayesian phylogenetics analysis and “relaxed” molecular dating estimation [31]. The data set was partitioned by codon and the GTR+I+Γ model was selected as the best evolutionary substitution model by likelihood-ratio test in MrModeltest v2.3 [32], [33]. We chose *BEAST model as our tree prior in BEAST analyses [34]. The analyses consisted of a random generated starting tree, uncorrelated lognormal relaxed molecular clock, and the program's default prior distributions of model parameters. Each analysis consisted of 40 million generations, sampled every 1,000 generations. The analyses were repeated four times and convergence was assessed using Tracer v1.5 [31]. Posterior probabilities (PP)≥0.95 are considered to be strongly or significantly supported [35].

Three divergence dates were used as the calibration points in molecular dating estimation and were treated as lognormal distributions [36] in analysis under a relaxed molecular clock model. This is the most appropriate method to use paleontological information [36] and overcomes the problem of rate variation across different timescale [37], [38]. The first calibration date was derived from *A. dominans*, the common ancestor of *A. flavicollis* and *A. sylvaticus* lived about 4.2–4.9 million years ago in European Mammal Neogene (MN) unit 14 [39]. In analysis, the mean sampled age was 4.2 Mya and the older 95% credible interval (CI) was 4.9 Mya (standard deviation = 0.1). The second calibration date was based on the oldest fossil of *Apodemus* that lived about 11.0–9.88 Mya in MN11 [40]. Estimation of divergence time was calculated with 9.88 Mya (offset = 9.0, mean = 0.88) as the mean sampled age and 11.0 Mya (standard deviation = 0.62) as the older 95% CI. The third calibration date was 12.3–11.0 Mya, the divergence time of *Mus* and *Rattus* [41]. The mean sampled age was 11.0 Mya (offset = 10.0, mean = 1.0) and the older 95% CI was 12.3 Mya (standard deviation = 0.63).

### Genetic diversity, structure, and population dynamics

The genetic distances among different evolutionary lineages and transversion/transition (Tv/Ts) ratios were estimated with MEGA v4.0 [26]. The number of haplotypes (N), nucleotide diversity (*pi*), and haplotype diversity (*Hd*) were calculated with DnaSP v5.10 [42]. The minimum-spanning networks were derived from Network v4.5 using the median-joining approach [43]. Because the Tv/Ts ratio was 5.33 in *A. ilex*, we set the weight of transversion to 5 and the weight of transition to 1 in analysis. Optimal phylogenetic trees were further calculated with the calculation options of maximum parsimony [44], [45].

Population subdivision was estimated using the hierarchical analysis of molecular variance (AMOVA) and Genetic differences among populations were calculated by pairwise *Fst* test using Arlequin v3.5 [46]. AMOVA were performed with 10,000 permutations with 3 different grouping options, which were grouped based either on the mtDNA clades/subclades identified in BEAST analysis or on their geographical distributions (see result). Pairwise *Fst* values were also performed with 10,000 permutations with a significant level of 0.05. An *Fst* value≥0.25 indicated that the gene flow was limited between two populations [47]. The historical population dynamics were analyzed by mismatch distribution analyses (MDA) [48], Fu's test [49] and Ramos-Onsins and Rozas's R2 test [50]. The behavior of *Fs* is better for large population sizes, whereas R2 is better for small sample sizes [50]. MDA and the Fu's test was performed using Arlequin v3.5 [46] with 10,000 permutations on each subclade or subgroup if neutrality holds statistical significance. The raggedness index (*rg*) and sum of squared deviations (SSD) between observed and expected mismatch distributions were estimated simultaneously with MDA. Under population expansion model, the *rg* and SSD were expected to have lower value [51]. R2 test was also performed using DnaSP v5.10 [42] and significant level was estimated by coalescent simulations with 10,000 replicates. If sudden expansion model was not rejected, the expansion time was calculated with the equation τ = 2ut [48], where t was measured in generations (one year per generation for *A. ilex*), τ was calculated simultaneously with MDA, u was calculated using formula u = 2 µk, in which 2 µ was the mutation rate per nucleotide and k was the number of nucleotides (1,140 bp).

## Results

### Phylogenetic analysis and molecular divergence time

Because *A. draco* and *A. ilex* are morphologically indistinguishable, the sequences of the samples were identified based on the pairwise comparison with the sequences from the specimens collected at or near their type localities. The type locality for *A. draco* is located at Kuatum, Fujian, China, while the type locality for *A. ilex* is at Salween - Mekong divide (28°20′N) [20]. In analysis, the sequences determined from the topotype specimen of *A. draco* by Liu *et al.* [8] (Accession number : AY389009) and the specimen from near the type locality of *A. ilex* from Mt. Meili, China (28°23.8′N) were serve as the reference sequences for *A. draco* and *A. ilex*, respectively. Of the 203 sequences generated in this study, 6 were identified as *A. draco* and 197 as *A. ilex* (Tables S1, S2). Haplotype analysis of 201 cyt-*b* sequences of *A. ilex*, including 4 sequences from GenBank, identified 134 haplotypes. The new identified haplotypes were submitted to GenBank (Accession numbers: JF503102–JF503107 (*A. draco*) and JF503109–JF503198, JF503200–JF503228, JF503230–JF503240 (*A. ilex*)).

**Figure 2:**
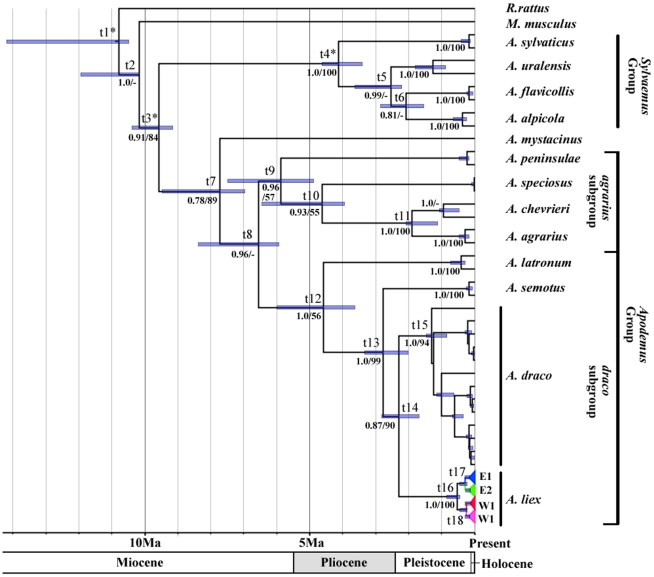
Chronogram of *Apodemus* based on cyt-*b* sequences. Branch lengths represent time; Node bars indicate the 95% CI for the clade age; An asterisk indicates node for calibration; The t_x_ above the nodes refer to median ages and 95% CI for each node in Table 2; Numbers below the nodes are Bayesian posterior probabilities (PP) and ML bootstrap (BS) values. A ‘-’ indicates the value is lower than 0.5 (PP) or 50 (BS).

**Figure 3:**
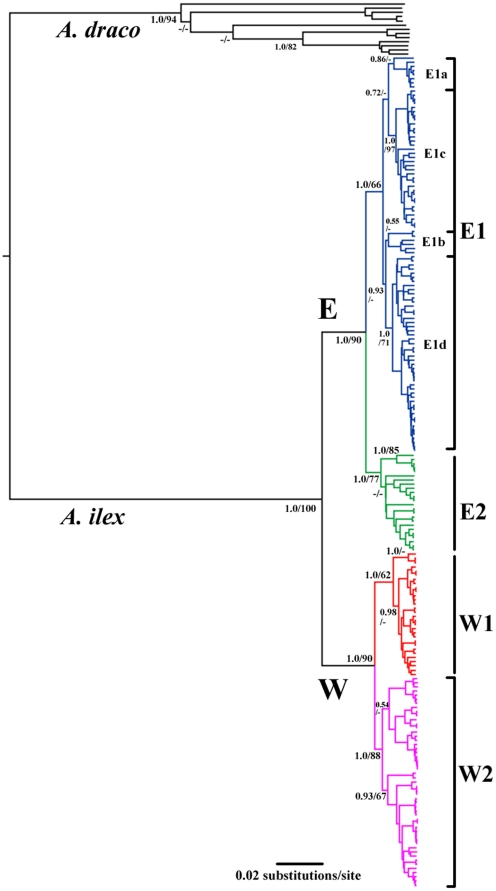
Bayesian phylogenetic analysis of *A. draco* and *A. ilex* based on cyt-*b* sequences. Branch lengths represent substitution per site and numbers at each node represent the Bayesian posterior probabilities and ML bootstrap values. A ‘-’ indicates the value is lower than 0.5 (PP) or 50 (BS).

The phylogeny estimated by RAxML and BEAST were congruent with each other and the topologies were overall highly supported. Thus, only the Bayesian trees were given and both Bayesian posterior probabilities and ML bootstrap support values (BS) were represented (Figures 2, 3). All populations of *A. ilex* and *A. draco* formed strongly supported (BS≥94, PP = 1.0) reciprocal monophyletic groups. The sister relationship between *A. draco* and *A. ilex* was also supported (BS = 90, PP = 0.87), with 9.0% of a K2P distance. All 201 *A. ilex* samples were grouped into the Eastern (E) and Western (W) clades, each containing two subclades: E1/E2 and W1/W2 (Figures 2, 3). All clades and subclades were significantly supported by BI analyses (PP = 1.0) and at least moderately supported by ML analyses (BS≥62). The K2P distances between clades and subclades were: E/W = 3.1%, E1/E2 = 1.9% and W1/W2 = 1.6%.

Table 1 presents the divergence times based on the Bayesian relaxed molecular dating estimation. *A. ilex* and *A. draco* diverged from their common ancestor at about 2.25 Mya (95% CI = 1.69–2.82). The earliest split within *A. draco* occurred about 1.15 Mya (95% CI = 0.84–1.46), much earlier than the split of the clades E and W of *A. ilex* at about 0.62 Mya (95% CI = 0.44–0.84). The divergence times of subclades E1/E2 and W1/W2 were at about 0.33 Mya (95% CI = 0.23–0.45) and 0.32 Mya (95% CI = 0.22–0.45), respectively.

**Table 1:** Divergence information within and between groups of *Apodemus*.

Node	Age	95% CI range	Divergence Event
t1*	11.92	10.49–14.20	*Rattus*/*Mus*
t2*	10.84	10.18–11.95	*Mus*/*Apodemus*
t3	9.63	9.16–10.39	*Sylvaemus* Group/*Apodemus* Group
t4*	3.97	3.41–4.63	*sylvaemus*/(*alpicola+flavicollis+tralensis*)
t5	2.87	2.21–3.63	*tralensis*/(*alpicola+flavicollis*)
t6	2.20	1.55–2.85	*flavicollis/alpicola*
t7	8.35	6.98–9.48	*Apodemus Group/mystacinus*
t8	7.06	5.94–8.38	*agrarius* subgroup/*draco* subgroup
t9	6.12	4.89–7.49	*peninsulae/(agrarius+chevrieri+speciosus)*
t10	5.13	3.95–6.45	*speciosus/(agrarius+chevrieri)*
t11	1.56	1.12–2.08	*chevrieri/agrarius*
t12	4.74	3.64–5.99	*latronum/(draco+ilex+semotus)*
t13	2.67	2.02–3.34	*semotus(draco+ilex)*
t14	2.25	1.69–2.82	*draco/ilex*
t15	1.15	0.84–1.46	*draco* MRCA
**t16**	**0.62**	**0.44–084**	***ilex*** ** E/W**
**t17**	**0.33**	**0.23–0.45**	***ilex*** ** E1/E2**
**t18**	**0.32**	**0.22–0.45**	***ilex*** ** W1/W2**

*Nodes used for calibration.

### Genetic diversity and structure of *A. ilex*

Phylogenetics analysis of 201 cyt-*b* sequences of *A. ilex* detected 965 conserved sites (84.6% of all sites) and 175 variable sites (15.4% of all sites). The K2P distances between haplotypes of *A. ilex* ranged from 0.0% to 4.1% (average 2.1%). The overall haplotype diversity (*Hd*) and nucleotide diversity (*Pi*) were 0.993 and 0.021, respectively. The pairwise *Fst* estimation among populations ranged widely from 0.00 to 1.00. Most populations are strongly differentiated from each other (*Fst*>0.25) indicating restricted gene flow. High levels of gene flow are more often observed within geographically close populations (e.g. population 16 and 18, *Fst*<0.001; Table S3).

In clade E, the haplotypes in both E1 and E2 are widely distributed in the east and some areas of the west of the Mekong River (populations 1, 14, 34, 36, 39 containing E1 haplotypes and population 1 containing E2 haplotypes; Figure 1). In clade W, the haplotypes in W1 were mainly distributed in the west of the Salween River and two localities east of the Mekong River (populations 10 and 26); while the haplotypes in W2 were distributed mainly in the southern part of the Yunnan-Guizhou Plateau and west of the Mekong River. In addition, sympatric distribution of different maternal lineages were observed in several localities (i.e. population 1, 2, 8, 10, 11, 16, 19, 34, 36, 39; Figure 1)

Further geographical structure was examined with AMOVA using three grouping options, including (1) the populations grouped by the subclades E1, E2, W1 and W2; (2) the populations grouped by geographical distributions, namely, the individuals from the west of the Salween River as group 1, the individuals distributed between the Salween and Mekong rivers as group 2, and the individuals from the east of the Mekong River as group 3; and (3) the populations grouped with the same way as (2) except for populations 1 and 14 which were included in group 3 (Figure 1). The results of AMOVA showed significant genetic structures at all hierarchical levels (P<0.001) and the largest proportion of variances were always found among groups (Table 2). In size order, the variances among groups were the second grouping option (43.30%)<the third grouping option (54.12%)<the first grouping option (55.17%), with the corresponding increased Φst values from 0.715, 0.737 to 0.822, respectively.

**Table 2:** Results of AMOVA based on different grouping options.

Groups	Φ_ST_	Φ_SC_	Φ_CT_	%among groups	%among populations within groups	%within populations
Four subclades	0.822*	0.603*	0.551*	55.17	27.05	17.78
Divided by two river	0.715*	0.497*	0.433*	43.30	28.20	28.50
Third choice	0.737*	0.427*	0.541*	54.12	19.57	26.31

*P<0.05.

**Figure 4:**
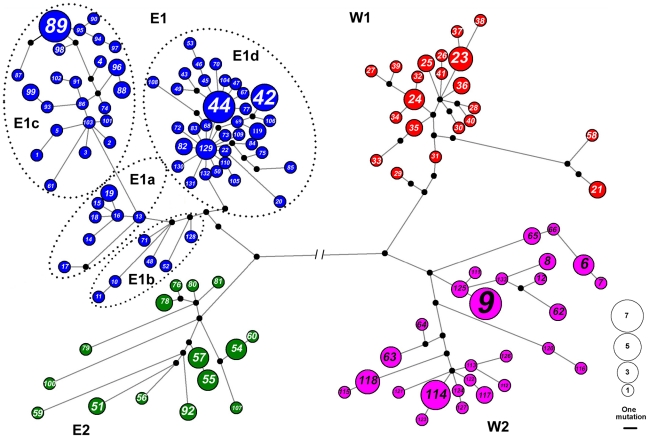
The median-joining network of *A. ilex* based on cyt-*b* sequences. The circle size is proportional to the haplotype frequency and the branch length is proportional to the number of mutations.

The network analyses generated eleven most parsimony trees that were similar to the gene tree inferred from the BEAST analyses, including four subclades (E1, E2, E3 and E4; Figure 4). The clade E1 has the most complex structure and can be further divided into 4 subgroups (E1a, E1b, E1c, and E1d). E1a consisted of the haplotypes from Caojian (population 14), E1b consisted of the haplotypes from Mt. Haba, Mt. Yulong, Lushi and Mt. Bangma (populations 3 and 6, 10, 24 and 36, respectively), E1c mainly included the haplotypes from the Mt. Wuliang and Mt. Ailao in central Yunnan (populations 15–22), and E1d contained the haplotypes from the northwest of Yunnan (Table S1). Star-like structures were found in E1c, E1d, W1 and W2 (Figure 4), though the original haplotypes were not found in W1 and W2. These structures are evidence of population expansion [52].

### Population historical demography of *A. ilex*

The analysis of MDA suggested that all four groups showed multimodal distributions (Figure S1) but with small SSD and rg values (Table 3). Moreover, Fu's and R2 test showed the large populations have significant negative *Fs* (i.e. E1, W1, W2) when small populations have significant small R2 (i.e. E2) (Table 3). Further analyses of the subgroups E1c and E1d revealed smooth unimodal mismatch distributions and significant negative Fu's *Fs* values (Table 3, Figure S1). The insignificant values of P_SSD_ and P_rg_ (P>0.05) in E1c, E1d and W2 confirmed the expansions within these group/subgroups; while the significant P_SSD_/P_rg_ (P<0.05) of W1 might be due to insufficient sample size [53]. Group E2 had multimodal distribution and insignificant negative Fu's *Fs* value, but the significant small R2 value and the insignificant values of P_SSD_ and P_rg_ implied the possibility of expansion in congruent with the network topologies. Because the divergence time between group E and W was at about 0.62 Mya, the population expansions of E1c, E1d and W1 were approximately at 0.064, 0.038 and 0.039 Mya, respectively (Table 3).

**Table 3:** Neutrality test and Mismatch distribution analyses of *A. ilex*.

Clade/group (sample size)	Neutrality test					Mismatch distribution analyses				
	Fu's *Fs*	P_Fs_	R2	P_R2_	SSD	P_SSD_	*rg*	P*_rg_*	Tau (95%CI)	expansion time (95%CI) (Mya)
E1 (94)	−24.37	<0.005	0.055	0.061	0.009	0.306	0.003	0.899	3.172 (1.025–18.281)	-
E2 (24)	−1.900	0.220	0.076	0.033	0.009	0.295	0.020	0.350	8.232 (5.523–13.916)	-
W1 (30)	−9.664	<0.005	0.081	0.119	0.025	0.016	0.048	0.045	4.389 (3.172–5.389)	0.039 (0.028–0.047)
W2 (50)	−5.920	0.062	0.088	0.305	0.006	0.467	0.010	0.527	12.348 (7.621–16.092)	-
E1c (32)	−12.155	<0.005	0.080	0.121	0.004	0.746	0.010	0.905	7.343 (4.045–10.094)	0.064 (0.035–0.089)
E1d (48)	−25.810	<0.005	0.038	0.002	<0.001	0.988	0.013	0.827	4.335(2.672–5.660)	0.038 (0.023–0.049)

## Discussion

### Taxonomic implication of *Apodemus ilex*

The taxonomic status of *A. draco*, *A. ilex* and *A. orestes* have been controversial for a long time. *Apodemus ilex* was named based on specimens collected from the Salween-Mekong divide (28°20″N), China [20] but was treated as a synonym of *A. orestes* [7] or *A. draco* [5], [54]. Musser et al. [6] included *orestes* within *A. draco*, but, after comparing the cranial characteristics between *A. orestes* and *A. draco* that are distributed in Wuliang Mountain, China, Jiang and Wang considered *A. orestes* as a valid species [3]. Patterns of genetic variations observed in the complex of *A. draco* based on cyt-*b* genes suggested that *A. orestes* was a subspecies of *A. draco*, and *A. ilex*, which is distributed in the Yunnan-Guizhou Plateau, is a valid species [8]. However, Musser et al. [2] still considered *A. ilex* and *A. orestes* as synonyms of *A. draco*.

With inclusion of 214 samples of *A. draco*/*ilex* that were widespread in the southern Hengduan Mountains and Yunnan-Guizhou Plateau, two major phylogroups were identified within the *A. draco* complex, one representing *A. draco* that consists of the specimens from eastern and western China, including all specimens from the western Sichuan Plateau, and another representing *A. ilex* that contains the samples mainly from the southern Hengduan Mountains and the western Yunnan Plateau. The average genetic distance between *A. draco* and *A. ilex* was 0.09 (K2P). The molecular dating estimation suggested that the divergence between *A. draco* and *A. ilex* was at about 2.25 Mya, earlier than the split of *A. alpicola* and *A. flavicollis* or *A. agrarius* and *A. chevrieri* at 2.20–1.56 Mya (Table 1, Figure 2). These results support the recognition of *A. ilex* is a valid species under genetic and phylogenetic species concepts [55], [56]. The taxonomic status of *A. orestes* will be discussed elsewhere (Chen *et al.* in preparation).

### Phylogeographic structure in *A. ilex* and topography of mountains and rivers

The Hengduan Mountains have long been recognized as a refugial area for animals [57], [58]. Previous analyses either focused on the northern Hengduan Mountain [59], [60], [61] or treated this area as one refugium Only a few studies have addressed the effect of the extremely complex topography of the southern Hengduan Mountains and the Yunnan Plateau [62], [63]. Our research revealed the significant internal genetic structure within the mountains which is relevant to the “microrefugia” [64] or “refugia within refugia” [65], [66]. These concepts are usually used to explain the phylogenetic structure in the refugia such as the Iberian Peninsula or disjunctive populations surviving in isolated microhabitats. The extremely complex topography [67], climate [68] and habitats [69] in the mountains as well as the mid-high elevation distributed pattern of *A. ilex* could have lead to the geographically isolation of *A.ilex* among different mountain areas and the subsequently restricted gene flow, which are respond for the strong geographic structure and the high pairwise *F*st values [70].

The minimum-spanning network and AMOVA analyses indicated the geographic structure of *A. ilex* was also shaped by the Mekong and Salween river systems. When the two rivers were setup as the genetic barriers in the AMOVA analyses, the variances existed mainly among populations in different regions (Φ_ST_ = 0.715) (Table 2). This result is congruent with the recent proposed hypothesis that deep river valleys may have acted as barriers to *Apodemus* species [1] as well as to other animals [71]. However, another montane mammal, the Yunnan hare (*Lepus comus*) has no phylogeographic pattern in the same area. Neither the area of low-elevation nor the river systems seem to be the barrier for the hares [72]. On the other hand, the paleo-drainage systems have facilitated dispersal of a frog species [62]. The discrepancy may due to the different habitats, dispersal abilities and colonization histories. The *Nanorana yunnanensis* is a semi-aquatic anuran living in cold montane streams [62]; the *L. comus* is much larger than *A. ilex* and may have colonized this area only recently [72].

### Effect of glaciation

The biogeographic histories of the montane inhabitants are usually affected by Pleistocene glacial cycles [73], [74], [75]. Generally, animals respond to climate change in two different ways [76]. First is by changing their distribution. The montane animals usually have larger distribution during glacial periods (but see [77], [78]) when they spread to lowland [79], [80], and the glacial and interglacial climate fluctuation can result in population isolation and reconnection [73], [74], [81], [82], [83], [84]. Second is by adapting to new environments [75], [76], [81]. Apparently, *A. ilex* occupies the same habitats as its relatives (i.e. *A. draco* and *A. semotus*) and hasn't adapted to a new environment, thus it had to shift distribution responding to climate change.

With a Bayesian method under a “relaxed” clock model, *A. ilex* diverged from *A. draco* at around 2.25 Mya. Therefore, the ancestor of *A. ilex* probably expanded southward from the northern Hengduan Mountains during global cooling in the period 2.7–2.5 Mya [85]. After that, divergence of *A. draco/ilex* may be attributed to the accelerated uplift of the Qinghai-Tibet Plateau and the resulting geomorphic changes of the plateau and the surrounding areas [86] as well as to turnover of vegetation and habitats [87]. The divergence between the eastern and western populations of *A. ilex* (∼0.62 Mya) was within the Yulong glaciation (0.73–0.5 Mya) [88] and the simultaneous divergences of the subclades E1/E2 (0.33 Mya) and W1/W2 (0.32 Mya) were consistent with the Lijiang glaciation (0.31–0.13 Mya) [89]. Because the calibration points we used are old (4 Mya to 12 Mya), these divergence time should be taken with caution. However, if the divergence time are “true”, they provide evidence that “nunatak refugia” existed in these mountains. Nunataks are refugia in mountain ranges above the glaciers and are snow-free during glaciations [78]. Indeed, there are evidence of valley glaciers in the Hengduan Mountains [89], [90]. Accordingly, *A. ilex* probably widely colonized the southern Hengduan Mountains and the Yunnan Plateau before the Yulong glaciation. The development of valley glaciers during the Yulong and Lijiang glaciation resulted in geographical isolation but the populations survived in multiple *in situ* refugia [77], [78] which result in divergences of the clades/subclades.

Sympatric distributions of different lineages are observed in several localities (Figure 1) which may be attributed to population expansion and the resulting secondary contact during climate fluctuations as well as to the complicated geological history of the drainage system [91]. Both the neutral test and MDA analyses provided evidence of expansion in each clade/subclade. The expansions of E1c, E1d and W1were roughly from 0.089 Mya to 0.023 Mya, corresponding to the last glacial period since 0.11 Mya [92]. Clade W1 and W2 are well segregated by the Salween rivers with only a few exception. On the other hand, without major physical barriers in the east of the Mekong River, populations can expand much more easily and colonize new habitats, resulting in secondary contact of E1 and E2.

### Conclusion and Perspectives

The Hengduan Mountains are the most important refugial region in China. Other studies have regarded the mountains as a single refugium, our research has, however, revealed significant internal genetic structure which suggests that the “microrefugia” or “refugia within refugia” models are more relevant.

Our finding suggests that both the low-elevation areas and deep river valleys are strong geographic barriers for *A.ilex*. However, for aquatic animals in this area, the drainage system is more likely to facilitate dispersal rather than prohibit it [62]. Thus, it seems the drainage system did play a role in shaping geographic patterns, but in different ways for different animals. Furthermore, the evolution of the drainage system may have led to a more complex geographic pattern.

Paleoclimatic change has also shaped genetic structure. The glacial-interglacial cycles not only resulted in inter- and intraspecific divergence, but also led to population expansion and secondary contact.

Our study has shed light on the biodiversity in this area. However, because of the complex topography of the mountains, complicated geological history of the drainage system, Pleistocene climate fluctuation and habitat turnover, it is far from clear how the high endemic biodiversity came into existence. It would be necessary to use comparative phylogeographic approaches of animals distributed in different habitats and with different dispersal abilities to examine how the topography, geographic events and climate change have shaped the biodiversity in the mountains of Southwest China [74], [93], [94].

## Supporting Information

Figure S1 **MDA and Fu's** ***Fs*** **test for four subclades of** ***A. ilex*** **.**
(TIF)

Table S1 Sampling information and genetic variability of *A. draco/ilex* used in this study.
(DOC)

Table S2 Information of outgroups used in this study.
(DOC)

Table S3 Pairwise FST values for all pair of populations.
(XLS)
