# Supplementary figures and images for: Phylogeographic Study of Apodemus ilex (Rodentia: Muridae) in Southwest China
Source: PLoS One. 2012 Feb 7;7(2):e31453. doi: 10.1371/journal.pone.0031453 (PMC3274519; doi:10.1371/journal.pone.0031453)

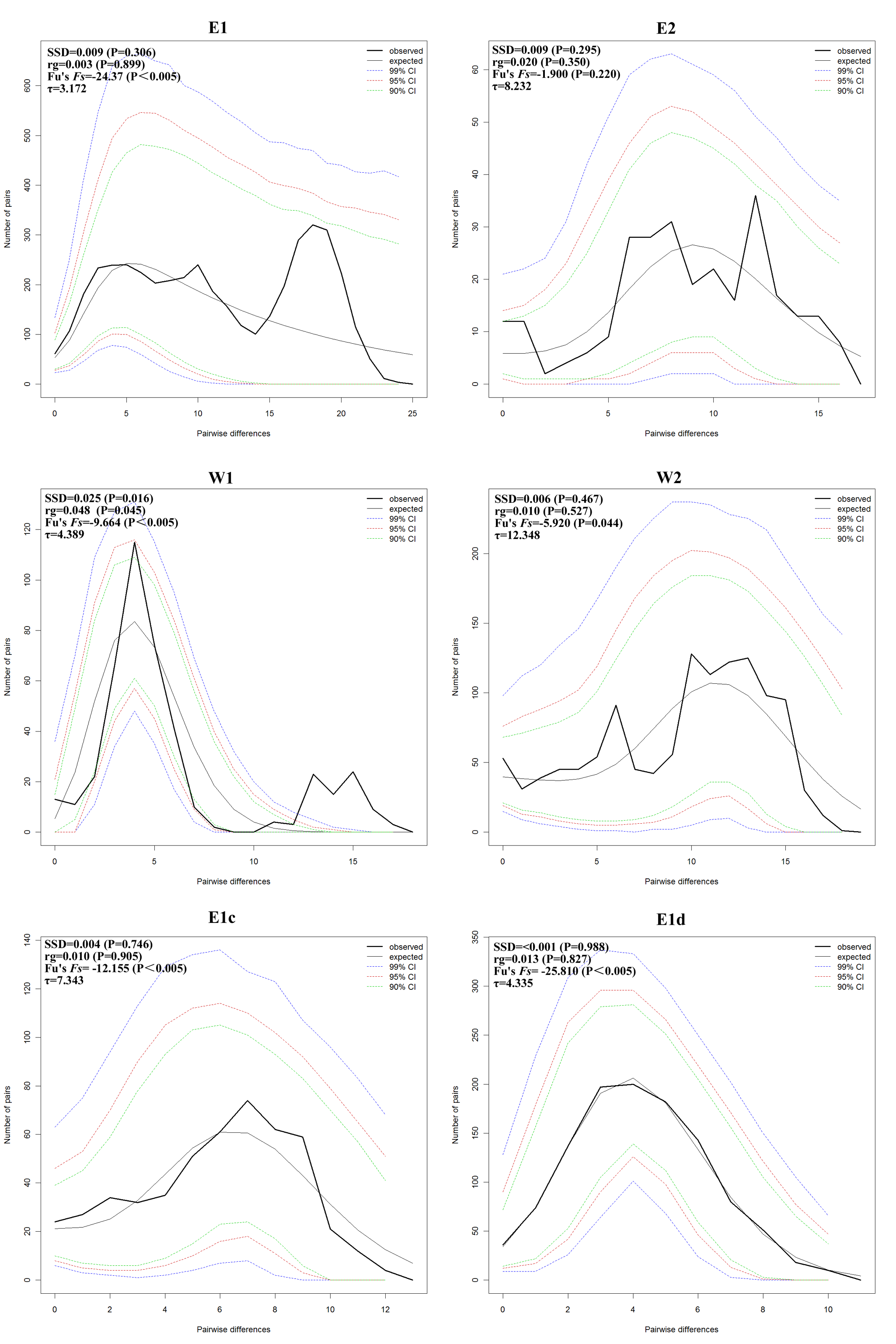

Supplement: Figure S1 — MDA and Fu's Fs test for four subclades of A. ilex . (TIF) [file pone.0031453.s001.tif]
