# Supplementary material for: Phylogeographic Study of Apodemus ilex (Rodentia: Muridae) in Southwest China
Source: PLoS One. 2012 Feb 7;7(2):e31453. doi: 10.1371/journal.pone.0031453 (PMC3274519; doi:10.1371/journal.pone.0031453)
Supplement: Table S1 — Sampling information and genetic variability of A. draco/ilex used in this study. (DOC) [file pone.0031453.s002.doc]

Table S1. Sampling information and genetic variability of *A. draco/ilex* used in this study.

| Site NO | Locality | Sample code | ilex Lineage | Haplotype (numbers) | N | *Hd* ±SD | Pi±SD |
| --- | --- | --- | --- | --- | --- | --- | --- |
| 1 | Yubeng,Mt. Meili, YN | ML1-ML8 | E1d, E2, W2 | 76,77,78(2),79,80,81,9 | 8 | 0.964±0.077 | 0.012 ±0.007 |
| 2 | Ciguzuzu field, Mt. Haba, YN | HB1-HB5 | E1c, E1d, E2 | 5(2),49,50,51 | 5 | 0.900±0.161 | 0.011 ±0.007 |
| 3 | Qiaotou, Mt. Haba, YN | HB6-HB8 | E1b, E1d | 42,52,53 | 3 | 1.000±0.272 | 0.008±0.007 |
| 4 | Dajianshanping, Mt. Haba, YN | HB9 - HB12 | E1b | 42(2),43,44 | 4 | 0.833±0.222 | 0.004 ±0.003 |
| 5 | Daojiaoping, Mt. Haba, YN | HB13 - HB17 | E1d | 44(3),45,46 | 5 | 0.700±0.218 | 0.001 ±0.001 |
| 6 | Edi, Mt. Haba, YN | HB18 - HB19 | E1b | 47,48 | 2 | 1.000±0.500 | 0.009 ±0.009 |
| 7 | Daju, Mt. Yulong, YN | YL1 - YL3 | E1d | 119,129,130 | 3 | 1.000±0.272 | 0.003 ±0.003 |
| 8 | Maoniupinig, Mt. Yulong, YN | YL4 - YL5 | E1d, W2 | 117,44 | 2 | 1.000±0.500 | 0.026 ±0.027 |
| 9 | Ganhaizi, Mt. Yulong, YN | YL6 - YL9 | E1d | 131,132,129,44 | 4 | 1.000±0.177 | 0.002 ±0.002 |
| 10 | Yunshanping, Mt. Yulong, YN | YL10 - YL12 | E1b, E1d, W1 | 128,129,36 | 3 | 1.000±0.272 | 0.019 ±0.015 |
| 11 | Tacheng, Weixi, YN | WX1 - WX8 | E1d, E2 | 104-110,44 | 8 | 1.000±0.063 | 0.008 ±0.005 |
| 12 | Xinshengqiao, Lanping, YN | LP1 - LP4 | E1d | 67,68,69,70 | 4 | 1.000±0.177 | 0.004 ±0.003 |
| 13 | Nuodeng, Yunlong, YN | TC 1- TC5 | E1d | 82(2),83,84,85 | 5 | 0.900±0.161 | 0.003 ±0.002 |
| 14 | Caojian, Yunlong, YN | CJ 1- CJ8 | E1a | 13-18,19(2) | 8 | 0.964±0.077 | 0.003 ±0.002 |
| 15 | Xujiaba, Mt. Ailao, YN | AL1 - AL5 | E1c | 1,2,3,4,5 | 5 | 1.000±0.127 | 0.004 ±0.003 |
| 16 | Shale, Nanjian, YN | WL1 - WL5 | E1c, E2 | 86,93,99,100,101 | 5 | 1.000±0.127 | 0.010 ±0.006 |
| 17 | Baohua, Nanjian, YN | WL6 - WL9 | E1c | 102,103,89,99 | 4 | 1.000 ±0.177 | 0.005 ± 0.004 |
| 18 | Modaohe, Jindong, YN | WL10 - WL14 | E1c | 87,88,89(2),90 | 5 | 0.900 ±0.161 | 0.004 ± 0.003 |
| 19 | Wangjiajing, Jindong, YN | WL15 - WL19 | E1c, E2 | 91,92(2),89,88 | 5 | 0.900 ±0.161 | 0.015 ± 0.009 |
| 20 | Raomalu, Jindong, YN | WL20 - WL21 | E1c | 89(2) | 2 | 0.000 ±0.000 | 0.000 ± 0.000 |
| 21 | Dazhaizi, Jindong, YN | WL22 - WL23 | E1c | 94,95 | 2 | 1.000 ±0.500 | 0.001 ± 0.001 |
| 22 | Huangcaoling, Jindong, YN | WL24 - WL28 | E1c | 96(2),97,98,89 | 5 | 0.900 ±0.161 | 0.004 ± 0.003 |
| 23 | Luodang, Fengqing, YN | LD1 - LD3 | W2 | 65(2),66 | 3 | 0.667 ± 0.314 | 0.001 ± 0.001 |
| 24 | Lushi, Fengqing, YN | LS1 - LS5 | E1b, E1c, E1d | 71,72,73,74,75 | 5 | 1.000±0.127 | 0.009 ± 0.006 |
| 25 | Wumeng, Luquan, YN | JZ1 - JZ2, JZ4 -JZ5 | E2 | 54,55,59,60 | 4 | 1.000±0.177 | 0.009 ±0.006 |
| 26 | Fenghuangchang, Mt. Jiaozi, YN | JZ3 | W1 | 58 | 1 |  |  |
| 27 | Hongtudi, Mt. Jiaozi, YN | JZ6 - JZ14 | E2 | 55(2),51,57(3),54(2),56 | 9 | 0.861±0.087 | 0.005 ±0.003 |
| 28 | 48daohe, Yongde, YN | YD1 - YD5 | W2 | 111,118,114,123124 | 5 | 1.000±0.127 | 0.006 ±0.004 |
| 29 | Zhongjiaochang, Yongde, YN | YD6 - YD7 | W2 | 125,126 | 2 | 1.000±0.500 | 0.001 ±0.010 |
| 30 | Taojin river, Yongde, YN | YD8 - YD10 | W2 | 127,118,112 | 3 | 1.000±0.272 | 0.005 ±0.004 |
| 31 | Yangwanshun, Yongde, YN | YD11 - YD14 | W2 | 113,114,115,116 | 4 | 1.000±0.177 | 0.009 ±0.006 |
| 32 | Xiaoxueshan, Yongde, YN | YD15, YD24,YD25 | W2 | 114,122,114 | 3 | 0.667±0.314 | 0.001 ±0.001 |
| 33 | Yinchangjie,Yondde, YN | YD16 - YD18 | W2 | 117,118(2) | 3 | 0.667±0.314 | 0.004 ±0.003 |
| 34 | Ganhe, Yongde, YN | YD19 - YD23 | E1d, W2 | 114(2),119,120,121 | 5 | 0.900±0.161 | 0.015 ±0.009 |
| 35 | Bangdong, Lincang, YN | LC1 - LC6 | W2 | 62(2),63(3),64 | 6 | 0.733±0.155 | 0.007 ±0.004 |
| 36 | Mengku, Mt. Bangma, YN | BM1 - BM7 | E1b, W2 | 6(3),9,8,10,11 | 7 | 0.857±0.137 | 0.019 ±0.011 |
| 37 | Xiaobangma, Mt. Bangma, YN | BM8 - BM12 | W2 | 9(2),12,7,8 | 5 | 0.900±0.161 | 0.007 ±0.004 |
| 38 | Dabangma, Mt. Bangma, YN | BM13 - BM14 | W2 | 9(2) | 2 | 0.000±0.000 | 0.000 ±0.000 |
| 39 | Dizhengdang, Gongshan, YN | DZD1 - DZD5 | E1d, W1 | 20,21(2),22,23 | 5 | 0.900±0.161 | 0.021 ±0.013 |
| 40 | Changlangba, Yunxian, YN | YX1 - YX2 | W2 | 125,9 | 2 | 1.000±0.500 | 0.001 ±0.001 |
| 41 | Dazhaoshan, Yunxian, YN | YX3 | W2 | 133 | 1 |  |  |
| 42 | Baihualing, Baoshan, YN | GLGS1 - GLGS5 | W1 | 24(3),35,39 | 5 | 0.700 ± 0.218 | 0.001 ± 0.001 |
| 43 | Pianma, Lushui, YN | GLGS6 - GLGS9 | W1 | 25,40,41,35 | 4 | 1.000±0.177 | 0.003 ± 0.002 |
| 44 | Yaojiaping, Tengchong, YN | GLGS10 - GLGS12 | W1 | 25,26,27 | 3 | 1.000±0.272 | 0.005 ± 0.004 |
| 45 | Shibali, Mt. Gaoligong, YN | GLGS13- GLGS15 | W1 | 28,29,30 | 3 | 1.000±0.272 | 0.004 ± 0.003 |
| 46 | Mingguang, Tengcheng, YN | GLGS16 - GLGS20 | W1 | 31,32,33,34,36 | 5 | 1.000±0.127 | 0.004 ± 0.003 |
| 47 | Liangshan, Longlin, YN | GLGS21 - GLGS25 | W1 | 37,23(3),38, | 5 | 0.700±0.218 | 0.001 ± 0.001 |
| 48 | Kunming, YN | KM (AB096825) | E1c | 61 | 1 |  |  |
| - | Mt. Ailao, YN | AL0 (AY389017) | E1 |  |  |  |  |
| - | Daxueshan, YN | YD0 (AY389018) | W2 |  |  |  |  |
| - | Mt. Wuliang, YN | WL0 (AY389019) | E1 |  |  |  |  |
| 49 | Yuexi, SC | A. draco8 - A. draco9 |  |  |  |  |  |
| 50 | Shimian, SC | A. draco10 - A. draco11 |  |  |  |  |  |
| 51 | Baoxing, SC | A. draco12 - A. draco13 |  |  |  |  |  |
| - | Fujian, China | A. draco1 (AY389009) |  |  |  |  |  |
| - | Sichuan, China | A. draco2 (AY389007) |  |  |  |  |  |
| - | Shanxi, China | A. draco3 (AY389004) |  |  |  |  |  |
| - | Sichuan, China | A. draco4 (AY389010) |  |  |  |  |  |
| - | Anhui, China | A. draco5 (AY389008) |  |  |  |  |  |
| - | Beijing, China | A. draco6 (AY389006) |  |  |  |  |  |
| - | Hebei, China | A. draco7 (AY389005) |  |  |  |  |  |

Sample size (N), haplotypes, haplotype diversity (*Hd*) and nucleotide diversity (*pi*) of each population of *A. ilex* are presented. YN: Yunnan province of China. SC: Sichuan Province of China.
