# Supplementary material for: Phylogeographic Study of Apodemus ilex (Rodentia: Muridae) in Southwest China
Source: PLoS One. 2012 Feb 7;7(2):e31453. doi: 10.1371/journal.pone.0031453 (PMC3274519; doi:10.1371/journal.pone.0031453)
Supplement: Table S2 — Information of outgroups used in this study. (DOC) [file pone.0031453.s003.doc]

Table S2. Information of outgroups used in this study.

| Species | Sampling locality | Code | Accession No. |
| --- | --- | --- | --- |
| *A. semotus* | Taiwan, China | A. semotus1 | AB033694 * |
|  | Taiwan, China | A. semotus2 | EU349734 * |
| *A. agrarius* | Shandong, China | A. agrarius1 | AY389012 * |
|  | Shaanxi, China | A. agrarius2 | AY389011 * |
| *A. alpicola* | Stuben, Austria | A. alpicola1 | AB032854 * |
|  | Switzerland | A. alpicola2 | AF159391 * |
| *A. chevrieri* | Sichuan, China | A. chevrieri1 | AY389015 * |
|  | Yunnan, China | A. chevrieri2 | JF7961111 |
| *A. draco* | Fujian, China | A. draco1 | AY389009 * |
|  | Sichuan, China | A. draco2 | AY389007 * |
|  | Shanxi, China | A. draco3 | AY389004 * |
|  | Sichuan, China | A. draco4 | AY389010 * |
|  | Anhui, China | A. draco5 | AY389008 * |
|  | Beijing, China | A. draco6 | AY389006 * |
|  | Hebei, China | A. draco7 | AY389005 * |
|  | Yuexi, Sichuan, China | A. draco8  A. draco9 | JF503102  JF503103 |
|  | Shimian, Sichuan, China | A. draco10  A. draco11 | JF503104  JF503105 |
|  | Baoxing, Sichuan, China | A. draco12  A. draco13 | JF503106  JF503107 |
| *A. flavicollis* | Konstanz,Germany | A. flavicollis1 | AF159392 * |
|  | Champer,Swiss | A. flavicollis2 | AB032853 * |
| *A. latronum* | Sichuan, China | A. latronum1 | AY389020 * |
|  | Yunnan, China | A. latronum2 | JF796110 |
| *A. mystacinus* | - | A. mystacinus | AF159394 * |
| *A. peninsulae* | Heilongjiang, China | A. Peninsulae1 | AY389000 * |
|  | Jilin, China | A. Peninsulae2 | AY388999 * |
| *A. specious* | Japan | A. specious1 | AB164483 * |
|  | Naganuma,Japan | A. specious2 | AB032849 * |
| *A. sylvaticus* | Leiden, Holland | A. Sylvaticus | AB033695 * |
| *A. uralensis* | Xinjiang, China | A. uralensis1 | AY389021 * |
|  | Kazakhstan | A. uralensis2 | AB096837 * |
|  | Pakistan | A. uralensis3 | AF160603 * |
| *Mus musculus* | - | Mus musculus | voo711 * |
| *Rattus rattus* | Miyazaki, Japan | Rattus rattus | AB033702 * |

* sequences from GenBank
